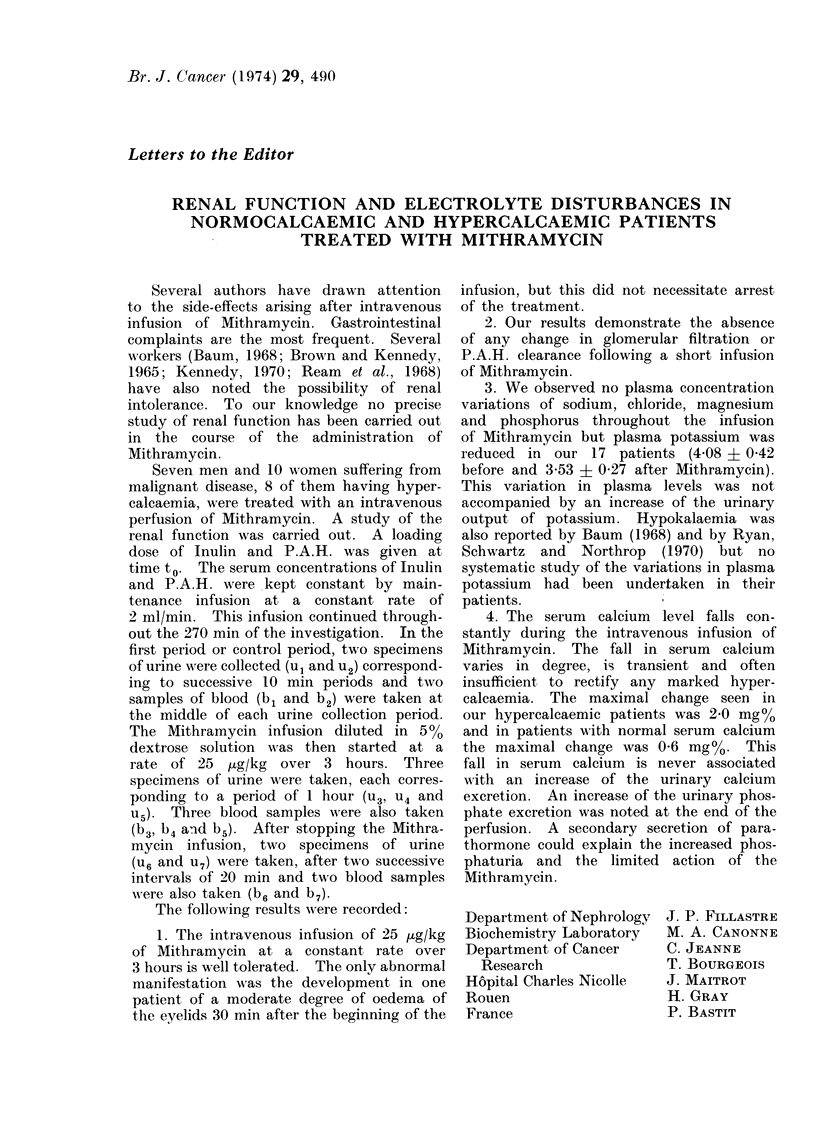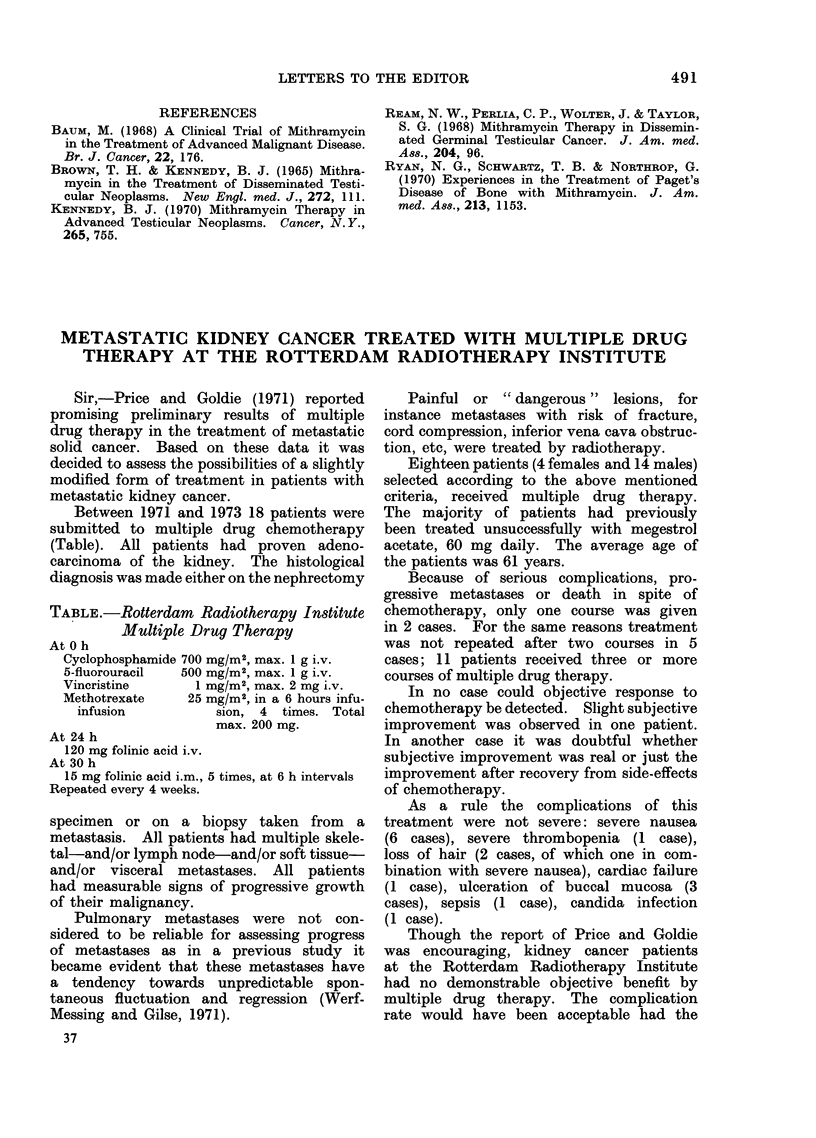# Letter: Renal function and electrolyte disturbances in normocalcaemic and hypercalcaemic patients treated with mithramycin.

**DOI:** 10.1038/bjc.1974.103

**Published:** 1974-06

**Authors:** J. P. Fillastre, M. A. Canonne, C. Jeanne, T. Bourgeois, J. Maitrot, H. Gray, P. Bastit


*Br. J. Cancer* (1974) **29**, 490

### *Letters to the Editor*

### RENAL FUNCTION AND ELECTROLYTE DISTURBANCES IN NORMOCALCAEMIC AND HYPERCALCAEMIC PATIENTS TREATED WITH MITHRAMYCIN

Several authors have drawn attention to the side-effects arising after intravenous infusion of Mithramycin. Gastrointestinal complaints are the most frequent. Several workers (Baum, 1968; Brown and Kennedy, 1965; Kennedy, 1970; Ream *et al.*, 1968) have also noted the possibility of renal intolerance. To our knowledge no precise study of renal function has been carried out in the course of the administration of Mithramycin.

Seven men and 10 women suffering from malignant disease, 8 of them having hypercalcaemia, were treated with an intravenous perfusion of Mithramycin. A study of the renal function was carried out. A loading dose of Inulin and P.A.H. was given at time $t_0$. The serum concentrations of Inulin and P.A.H. were kept constant by maintenance infusion at a constant rate of 2 ml/min. This infusion continued throughout the 270 min of the investigation. In the first period or control period, two specimens of urine were collected ($u_1$ and $u_2$) corresponding to successive 10 min periods and two samples of blood ($b_1$ and $b_2$) were taken at the middle of each urine collection period. The Mithramycin infusion diluted in 5% dextrose solution was then started at a rate of 25 $\mu$g/kg over 3 hours. Three specimens of urine were taken, each corresponding to a period of 1 hour ($u_3$, $u_4$ and $u_5$). Three blood samples were also taken ($b_3$, $b_4$ and $b_5$). After stopping the Mithramycin infusion, two specimens of urine ($u_6$ and $u_7$) were taken, after two successive intervals of 20 min and two blood samples were also taken ($b_6$ and $b_7$).

The following results were recorded:

1. The intravenous infusion of 25 $\mu$g/kg of Mithramycin at a constant rate over 3 hours is well tolerated. The only abnormal manifestation was the development in one patient of a moderate degree of oedema of the eyelids 30 min after the beginning of the infusion, but this did not necessitate arrest of the treatment.

2. Our results demonstrate the absence of any change in glomerular filtration or P.A.H. clearance following a short infusion of Mithramycin.

3. We observed no plasma concentration variations of sodium, chloride, magnesium and phosphorus throughout the infusion of Mithramycin but plasma potassium was reduced in our 17 patients ($4{\cdot}08 \pm 0{\cdot}42$ before and $3{\cdot}53 \pm 0{\cdot}27$ after Mithramycin). This variation in plasma levels was not accompanied by an increase of the urinary output of potassium. Hypokalaemia was also reported by Baum (1968) and by Ryan, Schwartz and Northrop (1970) but no systematic study of the variations in plasma potassium had been undertaken in their patients.

4. The serum calcium level falls constantly during the intravenous infusion of Mithramycin. The fall in serum calcium varies in degree, is transient and often insufficient to rectify any marked hypercalcaemia. The maximal change seen in our hypercalcaemic patients was 2·0 mg% and in patients with normal serum calcium the maximal change was 0·6 mg%. This fall in serum calcium is never associated with an increase of the urinary calcium excretion. An increase of the urinary phosphate excretion was noted at the end of the perfusion. A secondary secretion of parathormone could explain the increased phosphaturia and the limited action of the Mithramycin.

Department of Nephrology
Biochemistry Laboratory
Department of Cancer Research
Hôpital Charles Nicolle
Rouen
France

J. P. FILLASTRE
M. A. CANONNE
C. JEANNE
T. BOURGEOIS
J. MAITROT
H. GRAY
P. BASTIT

REFERENCES

BAUM, M. (1968) A Clinical Trial of Mithramycin in the Treatment of Advanced Malignant Disease. *Br. J. Cancer*, **22**, 176.

BROWN, T. H. & KENNEDY, B. J. (1965) Mithramycin in the Treatment of Disseminated Testicular Neoplasms. *New Engl. med. J.*, **272**, 111.

KENNEDY, B. J. (1970) Mithramycin Therapy in Advanced Testicular Neoplasms. *Cancer, N.Y.*, **265**, 755.

REAM, N. W., PERLIA, C. P., WOLTER, J. & TAYLOR, S. G. (1968) Mithramycin Therapy in Disseminated Germinal Testicular Cancer. *J. Am. med. Ass.*, **204**, 96.

RYAN, N. G., SCHWARTZ, T. B. & NORTHROP, G. (1970) Experiences in the Treatment of Paget's Disease of Bone with Mithramycin. *J. Am. med. Ass.*, **213**, 1153.